# Triple Combination of Immune Checkpoint Inhibitors and BRAF/MEK Inhibitors in *BRAF*V600 Melanoma: Current Status and Future Perspectives

**DOI:** 10.3390/cancers14225489

**Published:** 2022-11-08

**Authors:** Michèle Welti, Florentia Dimitriou, Ralf Gutzmer, Reinhard Dummer

**Affiliations:** 1 Faculty of Medicine, University of Zurich (UZH), 8006 Zurich, Switzerland; 2 Department of Dermatology, University Hospital Zurich (USZ), 8091 Zurich, Switzerland; 3 Department of Dermatology, Johannes Wesling Medical Center, Ruhr University Bochum, 32423 Minden, Germany

**Keywords:** triple therapy, metastatic melanoma, BRAF mutation, immune checkpoint inhibitor, targeted therapy

## Abstract

**Simple Summary:**

With the development of immunotherapies and targeted therapies in the last years, there has been great progress in the outcome of patients with metastatic melanoma. One of the current challenges is to optimize the treatment efficacy, to overcome resistance and to prevent disease relapses. This review analyzed the appropriate treatment sequence and the potential of the triple combination of immunotherapy and targeted therapy in *BRAF*V600-mutated melanoma. We summarized the results from phase 2 and 3 clinical trials investigating these treatment modalities in patients with advanced melanoma as well as in specific subpopulations in casu those with active brain metastases. We analyzed the study designs, the treatment efficacy in sequential treatment and in triple combination as well as the observed toxicity profile. In addition, we identified specific indications and limitations of triple combination in advanced *BRAF*V600 mutant melanoma.

**Abstract:**

Immune checkpoint inhibitors (ICIs), namely programmed cell death 1 (PD-1) or cytotoxic t-lymphocyte antigen 4 (CTLA-4) inhibitors, are currently the standard of care for the treatment of advanced melanoma, with robust and durable responses in a subset of patients. For BRAFV600-mutant melanoma, treatment with BRAF and MEK inhibitors has resulted in high objective response rates, but most responses are short-lived. Preclinical data suggest that BRAF and MEK inhibitors result in immunomodulatory changes in the tumor microenvironment; early data in murine models further suggest that these changes could enhance sensitivity to ICIs. Subsequently, the notion of combining the two therapy modalities for a more effective response was further evolved in early phase clinical trials. In this review, we analyzed the results of recent phase 2 and 3 clinical trials investigating the combination of ICIs with targeted therapy in BRAFV600-mutated advanced melanoma. Furthermore, we evaluated the results of recent studies investigating the first-line treatment sequencing of ipilimumab/nivolumab and BRAF/MEK inhibitors in these patients. We discussed the study limitations and interpreted how these recent advances could be incorporated into the treatment landscape of advanced BRAFV600-mutant melanoma.

## 1. Introduction

The advent of novel therapeutic options, including both immune checkpoint inhibitors (ICIs) and targeted therapies (TT), has resulted in remarkable advances in the treatment landscape of advanced melanoma [1,2,3,4]. Presently, in the first-line setting, the median overall survival (OS) of patients treated with combined programmed cell death 1 (PD-1) or cytotoxic T-lymphocyte antigen 4 (CTLA-4) inhibition has increased to 72.1 months in phase 3 clinical trials, and data on melanoma-specific survival (MSS) confirm the long-term treatment benefit of this treatment [5]. As such, monoclonal antibodies targeting either the PD-1 or the CTLA-4 protein are currently deemed as the standard of care in the treatment of advanced melanoma. Besides, in approximately 40–50% of melanomas, tumors are driven by oncogenic *BRAF* mutations [6]. This subgroup of patients can additionally benefit from treatment with BRAF/MEK inhibitors that target the mitogen-activated protein kinase (MAPK) pathway [7]. Despite the initial clinical benefit in the majority of the patients with high objective response rates (ORRs), most responses are short-lived, and acquired resistance is frequent [8]; approximately 70% of patients treated with dabrafenib and trametinib will experience a relapse within the first three years, and the median progression-free survival (mPFS) is 11.1 months [9]. Conversely, ICIs may result in robust and, in a subset of patients, durable disease remission, but only approximately 50% of patients will respond initially, which is indicative of higher rates of primary resistance [10]. Treatment strategies to improve outcomes aim to incorporate the benefits of both treatments in order to provide high response rates with a long-term benefit. In the present review, we evaluated the results of recent clinical trials investigating the treatment sequencing and the treatment combination of ICIs and TT in patients with advanced *BRAF*V600-mutant melanoma. We analyzed how these recent advances could be incorporated into the current management strategies of these patients, and we discussed the limitations and future perspectives.

### Preclinical Evidence for Improved Antitumor Activity of Immunotherapy with BRAF and MEK Inhibitors in BRAFV600 Melanoma

The potential role for the treatment combination of TT and ICIs has aimed to exploit the different kinetics of clinical response to the two drugs, resulting in optimized antitumor activity with high response rates and durability [11]. The hypothesis of a synergistic TT/ICI combination has been supported by preclinical data, indicating that the activation of the MAPK pathway leads to an immunosuppressive tumor microenvironment [11,12,13]. Precisely, the tumor microenvironment in *BRAF*-mutant melanoma is characterized by the enhanced expression of immunosuppressive cytokines, the decreased expression of melanoma differentiation antigens (MDAs) as well as human leukocyte antigens class I (HLA-I) and the suppressed the function of melanoma-specific cytotoxic cells [11]. BRAF inhibitors display immune sensitizing effects by increasing antigen presentation and antigen-specific T-cell recognition [14,15]. Additionally, tumor-infiltrating lymphocytes (TILs) increase early during the treatment course [16]. In clinical studies, the addition of a MEK inhibitor to the BRAF inhibitor treatment can reduce the toxicities related to paradoxical MAPK activation in BRAF wild-type cells and can additionally increase the treatment efficacy [7,17,18]. However, early evidence in preclinical data supports that, unlike the BRAF inhibitor, the MEK inhibitor has pleiotropic effects on tumor cells and lymphocytes. Although the impact of MEK inhibitors on T-cell differentiation is still unclear, preclinical studies support that MEK inhibitors may enhance the survival of activated T-cells but can have a negative impact on T-cell priming [19,20]. This interference with T-cell functions seems to be complex and context-dependent; further reports affirm the immunogenic effects of MEK inhibitors due to the upregulation of human leukocyte antigen (HLA) class I molecules and the reduction of immunosuppressive cytokines in the tumor microenvironment [14,15,21].

This preclinical evidence that both BRAF and MEK inhibitors can enhance the immune recognition of melanoma cells provides a strong rationale for the combination of immunotherapy and targeted therapy in advanced melanoma. Nevertheless, the initial attempts to combine a BRAF inhibitor (vemurafenib) with anti-CTLA-4 (ipilimumab) resulted in dose-limiting hepatic toxicity (DLT), leading to the discontinuation of this combination [22]. First investigated in murine models, BRAF and MEK inhibitors could mitigate paradoxical MAPK activation and could be efficiently combined with ICIs, whereas this triple combination also demonstrated some synergistic effects [23,24]. In one of these models, dabrafenib and trametinib in combination with pmel-1 adoptive cell transfer (ACT) improved antitumor activity by increasing T-cell infiltration into tumors and improving in vivo cytotoxicity [23]. On the other hand, the treatment sequencing of two doses of anti-PD-1 prior to MAPK-inhibitor combination maximized the antitumor efficacy and resulted in robust T-cell clonal expansion in both intracranial and extracranial tumor sites in a mouse model [25]. These results were further confirmed when a short-term BRAF/MEK inhibitor therapy was combined with anti-PD-1 [24], thus supporting the further investigation of this early evidence in phase 1 clinical trials.

## 2. Current Status

### 2.1. Published Studies in the First-Line Setting

Following early preclinical evidence, several phase 2 and 3 clinical trials have investigated the efficacy of triple combination with ICIs and BRAF/MEK inhibitors in patients with advanced *BRAF*V600-mutant melanoma. All treatments were conducted in first-line setting, and the choice of the control arm was based on the standard of care at the time of the study design; as such, the available data, except for the ongoing STARBOARD study [26], were compared to BRAF/MEK inhibitors in the control arm.

#### 2.1.1. Keynote-022 Study: Pembrolizumab, Dabrafenib and Trametinib

Initial data from a phase 2 clinical trial investigating the triple combination of anti-PD-1 with BRAF/MEK inhibitors in patients with BRAFV600 stage III and IV cutaneous melanoma were reported in June 2019 [27]. The Keynote-022 trial enrolled 120 patients that were randomly assigned 1:1 in the treatment group, treated with pembrolizumab, dabrafenib and trametinib, or the control group, treated with dabrafenib and trametinib. The primary endpoint of the study was the progression-free survival (PFS); secondary endpoints included the ORR, the duration of response (DoR) and the overall survival (OS). Baseline characteristics were overall well-balanced between the treatment arms, but in the triplet arm, there was a higher proportion of patients with M1c disease (82% vs. 63%), BRAFV600E mutation (86.7% vs. 81.7%) and metastases in more than two sites (60% vs. 51.7%). Of note, 13.3% of patients in the triplet arm had previously progressed in the adjuvant treatment. Initial results were analyzed after a median follow-up (mFU) of 9.6 months (range 2.7–23.4). Despite having a numerically longer PFS and DoR in the triple therapy, the results were not significant. In addition, the occurrence of grade 3–5 adverse events (AE) was much higher in the triplet group than in the control group (70% and 45%, respectively). After a longer follow-up, a clinical improvement in duration and survival was achieved in the triplet arm, although the primary endpoint of a statistically significant improvement on PFS was not met [28]. The latest results presented in 2022 showed that after an mFU of 61.2 months (range 50.7–67.5), the median PFS was 17 months (95% confidence interval (CI) 11.3 to not reached (NR)) in the triplet arm and 9.9 months (95% CI 6.7 to 15.6) in the doublet arm (hazard ratio (HR) 0.46; 95% CI 0.29 to 0.74) [29]. The median OS was 46.3 months (95% CI 23.9-NR) in the triplet arm and 26.3 months (95% CI 18.2–38.6) in the doublet arm. In the exploratory subgroup analysis for PFS, patients that were aged <65 years, male, had an eastern cooperative oncology group (ECOG) performance status (PS) of 0 and had lactate dehydrogenase (LDH) levels > the upper limit of normal (ULN) at baseline were more likely to profit from the triple therapy [28]. However, none of these factors were significant for a prolonged OS. Patients in the doublet arm were more likely to achieve an ORR (65% vs. 72%), but a complete response (CR) was more frequent in the triplet group (20% vs. 15%). The median duration of response (mDoR) was longer in the triplet arm compared to the doublet arm (30.2 months vs. 12.1 months; HR 0.32; 95% CI 0.17–0.59). Of note, despite the higher proportion of grade 3–5 immune-related adverse events (irAEs), drug exposure to BRAF/MEK inhibitors was higher in the triple group, compared to the double group (12.4 vs. 9.1 months).

#### 2.1.2. IMspire150 Study: Atezolizumab, Vemurafenib and Cobimetinib

The IMspire150 clinical trial was the first phase 3 study to investigate triple therapy in advanced stage IIIC-IV BRAFV600-mutant melanoma [30]. The study randomly assigned patients to receive a triple combination of the PD-L1 inhibitor atezolizumab with vemurafenib and cobimetinib versus vemurafenib and cobimetinib in a 1:1 randomization according to baseline LDH levels and geographical region. The treatment regimen was based on the previous results of the phase 1b study, in which the initial induction of vemurafenib and cobimetinib followed by the addition of atezolizumab after the first treatment cycle was tolerable and showed initial efficacy [27]. Of note, the addition of atezolizumab in the second treatment cycle was accompanied by a dose reduction of vemurafenib from 960 mg bid to 720 mg bid. Subsequently, a total of 514 treatment-naive patients were randomized in the phase 3 study and were included in the final analysis. The primary endpoint of the study was the investigator-assessed PFS. The study met its primary endpoint and was able to demonstrate an added value of atezolizumab to vemurafenib and cobimetinib. After an mFU of 18.9 months (interquartile range (IQR) 10.4–23.8 months), the investigator assessed that the PFS was 15.1 and 10.6 months (HR 0.78; *p* = 0.025) for the triple and doublet group, respectively. However, this difference was not significant when the PFS was assessed by an independent review (*p* = 0.16). The ORR was similar between the two groups (66.3% vs. 65%); same as the proportion of patients that achieved CR (15.7% vs. 17.1%). The mDoR was longer in patients treated with triple combination (21 vs. 12.6 month), and the PFS curves separated after seven months of treatment, indicating a prolonged treatment benefit with the addition of atezolizumab. The OS was not reported due to the short mFU. Updated results from a second interim analysis with a longer mFU did not show any statistical significance with regards to the OS [31]. Based on these results, triple therapy with atezolizumab, cobimetinib and vemurafenib received FDA approval; this combination is currently the only approved triple therapy.

#### 2.1.3. COMBI-i Study: Spartalizumab, Dabrafenib and Trametinib

In the phase 3 COMBI-i study, patients with advanced stage IIIC-IV cutaneous *BRAF*V600-mutant melanoma were randomly assigned to receive anti-PD-1 (spartalizumab) in combination with dabrafenib and trametinib, or dabrafenib/trametinib [32]. Details of the treatment regimen are shown in Table 1. The primary endpoint of the study was the investigator-assessed PFS. Secondary endpoints included the OS. The study did not meet the primary endpoint, but the results were in concordance to those previously reported in the Keynote-022 trial [33]. After an mFU of 27.2 months (IQR 25.4–29.0 months), the mPFS was 16.2 months (95% CI 12.7–23.9 months) in the triple group compared with 12.0 months (95% CI 10.2–15.4 months) in the double group (HR 0.82; 95% CI 0.66–1.03; *p* = 0.042; one-sided). In the subgroup analysis for the PFS, patients with ≥3 metastatic sites (*p* = 0.03) and a sum of lesion diameters ≥ 66 mm at baseline (*p* = 0.007) seemed to profit more from the triple therapy. The ORR did not differ significantly between the two treatment groups (69% vs. 64%), and the proportion of patients that achieved CR was also similar (20% vs. 18%). The mDoR was not reached (95% CI 18.6 months—NR) in the triple group, whereas the mDoR in the double group was 20.7 months (95% CI 13.0—NR). In the results from the landmark 3-year OS with a prolonged mFU of 42.8 months, the median overall survival (mOS) was not reached in the triple group, whereas in the double group, the mOS was 40.4 months (HR 0.79; 95% CI 0.62–1.03). The subgroup analysis showed an association of clinical factors, such as ECOG PS 1, age ≥ 65 years, negative PD-L1 status, sum of lesion diameters ≥ 66 mm at baseline and metastatic sites ≥ 3, with a prolonged OS in the triple group [34].

In this study, the increased toxicity in the triple combination arm was particularly evident. Treatment-related adverse events (trAEs) of any grade were observed in 88% of the patients in the control arm, with grade ≥ 3 AEs in 33% of the patients. This frequency was higher in the triple group with 99% of trAEs of any grade and 55% of grade ≥ 3. This toxicity profile resulted in dose modifications in 88% of the patients in the triple group compared with 73% in the double group.

#### 2.1.4. Toxicity Profiles

It is expected that, with the combined use of ICIs and BRAF/MEK inhibitors, the frequency of treatment-related side effects should increase accordingly. With BRAF/MEK inhibitors, side effects occurred in almost all patients (approx. 97%), and, although some of these were class effects, others might reflect differences in pharmacokinetic and pharmacodynamic characteristics [38]. On the contrary, the exact pathophysiology of irAEs due to ICIs is unknown, and, although most of them are usually reversible, they may rarely be associated with irreversible organ damage or even death [39]. Despite the substantial differences in their activity profiles, there is increasing evidence implying that the sequential combination of ICIs and targeted therapy might result in an exaggerated immune response, resulting in systemic symptoms similar to those observed in cytokine release syndrome (CRS) [40]. In the IMspire150 study, trAEs occurred in 99% of patients in the triple group and 99% of patients in the control group, whereas grade 3–4 trAEs were reported in 79% and 73% of the patients, respectively [30]. Of note, patients who discontinued the study after the run-in cycle with BRAF/MEK inhibitors due to AEs were considered as part of the control group in the safety analysis. Common grade 3–4 trAEs in the triple group included creatinine-kinase (CK) elevation (20%), alanine aminotransferase (ALT) elevation (13%), a maculopapular skin rash (13%), amylase elevation (10%) and aspartate aminotransferase (AST) elevation (8%). Treatment discontinuation occurred in 13% of the patients in the triple group compared with 16% in the control group. In the COMBI-i clinical trial, trAEs were reported in 99% of patients treated with spartalizumab, dabrafenib and trametinib and 88% in the control arm [32]. The rate of grade 3–4 trAEs was 55% and 33%, respectively. Common trAEs in the triple arm included pyrexia (66%), chills (29%), diarrhea (24%) and nausea (24%), whereas pyrexia (64%), chills (19.1%) and diarrhea (10.5%) were the most common AEs leading to dose modifications of any drug. Notably, 68% of patients in the triple arm, compared to 45% in the control arm, had at least one dose reduction. Permanent treatment discontinuation due to treatment intolerability was noted in 12% and 8% of patients, respectively. Similar to the COMBI-i clinical trial, grade 3–5 trAEs were reported in 58% of patients treated with pembrolizumab, dabrafenib and trametinib in the Keynote-022 study compared with 25% in the control arm, leading to dose interruption in 83% and 68% of the patients, respectively [33]. These trAEs are shown in Table 2.

### 2.2. Treatment Sequence and First-Line Treatment Selection in BRAFV600 Mutant Melanoma

The choice of the comparative arm in the above-mentioned studies has been controversially discussed since recent data largely favor first-line treatment with ICIs over BRAF/MEK inhibitors in *BRAF*V600-mutated melanoma [41]. Targeted therapy and immunotherapy show substantial differences in their kinetics of disease response and progression, such that, for many years, there was a dilemma over the appropriate sequence of these treatment modalities. Two randomized studies, the SECOMBIT and the DREAMseq clinical trials, were designed to better elucidate the optimal first-line treatment in patients with advanced BRAF*V600*-mutant melanoma.

The phase 2 SECOMBIT trial randomly assigned patients to encorafenib/binimetinib followed by ipilimumab/nivolumab upon disease progression (arm A), to the converse sequence (arm B) and to encorafenib/binimetinib for 8 weeks followed by a treatment switch to ipilimumab/nivolumab until disease progression (arm C) [42]. The primary endpoint of the study was the OS rate at two years, and the secondary endpoints included PFS and total PFS, defined as the time to second progression. Of note, the study was not powered to compare the three treatment arms. Despite the small study population, after an mFU of 32.2 months, the mOS was not reached in any of the study arms. However, the landmark 2-year OS rate was higher in patients of arm B (73%) compared to arm C (69%) and arm A (65%) [36]. The third arm of the study was of particular interest since it was based on translational data indicating that despite the immunomodulatory effects of BRAF and MEK inhibitors, these are in fact transient. Indeed, recent clinical and preclinical data support the theory that acquired resistance to targeted therapy can induce a cross-resistance to ICIs, initiated by an immunosuppressive tumor microenvironment that lacks functional dendritic cells [43]. Of note, patients treated in the third arm of the study demonstrated a clinical benefit but did not show any superior treatment responses compared to arm B. These survival data are in line with previous retrospective reports in real-life patients [44].

The DREAMseq clinical trial was similarly designed; patients were randomized to receive either ipilimumab/nivolumab (arm A) or dabrafenib/trametinib (arm B) with crossover to the alternate therapy at disease progression [45]. The primary endpoint was, accordingly, the landmark 2-year OS rate. In this study, the 2-year OS rate was 71.8% in the ipilimumab/nivolumab arm and 51.5% in patients starting with dabrafenib/trametinib (*p* = 0.01). This was analogous to the observed 2-year OS rate in the SECOMBIT study, although the second treatment arm showed a lower performance. In line with the previous data, treatment with ipilimumab/nivolumab after progression on first-line BRAF/MEK inhibitors resulted in lower response rates.

Nevertheless, both studies have crucial limitations: First, the SECOMBIT study was not powered to show the differences in the treatment efficacy of the three study arms. Second, both studies have been criticized for the exclusion of patients with symptomatic metastases, rapid disease progression or active brain metastases, all of which include real-life patients that would probably profit from a first-line induction course with BRAF/MEK inhibitors. However, both studies were designed in 2015, when little was known about the long-term response in patients treated with ICIs and targeted therapies. Lastly, the crossover design of the DREAMseq study and the protocol-required washout period in patients that progressed during ipilimumab/nivolumab excluded them from switching to second-line BRAF/MEK inhibitors. The crossover rate was only 52%, and 24 (18%) patients randomized in arm A died within the first 10 months of treatment, after receiving a median one cycle of ipilimumab/nivolumab. This underlines the limitations of a crossover study design; in real-life practice, *BRAF*V600-mutant patients would receive targeted therapy upon progression to ICIs, and in a context of a clinical trial, this probably excluded patients with aggressive tumor biology and rapid disease progression. Notably, patients with symptomatic metastases or with rapid disease progression might have profited from upfront BRAF/MEK inhibitor induction treatment, which is in agreement with arm C of the SECOMBIT study. However, the run-in phase with BRAF/MEK inhibitors should not be too long, as the interim analysis of the ImmunoCobiVem study, with a median follow-up of 19.0 months, showed that only a subset of patients responded to the therapy change to ICIs after the 3-month run-in with TT [46].

Taken together, today, first-line treatment with PD-1 based ICIs is the standard of care in inoperable melanoma. Thus, the question remains if the triple combination is superior to ICIs in the first-line setting. The STARBOARD clinical trial addresses this question. It is a phase 3 study that compares the efficacy and safety of the experimental group encorafenib/binimetinib with pembrolizumab versus pembrolizumab as a control group for the treatment of advanced *BRAF*V600E/K-mutant cutaneous melanoma (NCT04657991) [26].

### 2.3. Treatment Sequence and Triple Combination in the Neoadjuvant Treatment of Resectable BRAFV600-Mutant Stage III Melanoma

Recent studies suggest some major advantages in using neoadjuvant therapy in resectable stage III melanoma. Preclinical data showed that the presence of a clinical detectable tumor burden leads to an improved immune response to systemic therapy. The high tumor burden in the neoadjuvant setting seemed to result in beneficial antigen presentation and, thus, increased levels of tumor-specific CD8+ T cells [47,48,49,50]. In addition, a pathologic examination of the resected tumor provided morphological and immunological information about the response to the administered therapy [49]. Results from the OpACIN and OpACIN-neo trial, evaluating the efficiency, toxicity and dose finding of neoadjuvant ipilimumab plus nivolumab, supported the findings from the preclinical data [51,52]. The administration of two cycles of ipilimumab 1 mg bid and nivolumab 3 mg bid within 3 weeks led to a pathologic response rate of 77% (95% CI 58–90%) and grade 3–4 irAEs of 20%. The estimated 3-year relapse-free survival (RFS) was 79.3% (95% CI 65.9–95.5%), and the estimated 3-year OS was 93.3% (95% CI 84.8–100.0%), with the pathologic response remaining a surrogate marker for the RFS and the OS [53].

There were additional promising findings from recently presented trials in patients with resectable stage III or oligometastatic stage IV melanoma who received two doses nivolumab 480 mg and relatlimab 160 mg within 4 weeks of neoadjuvant therapy [50]. In this phase II trial with 30 patients, the overall pathologic response rate was 70%, and a pathologic complete response (pCR) was achieved in 57%. Patients with any pathologic response showed a 1- and 2-year RFS of 100% and 92% compared to 88% and 55% in patients without a pathologic response (*p* = 0.005). Neoadjuvant therapy using targeted therapy seems to also be a potential new treatment option for resectable stage III *BRAF*V600-mutant melanoma. In the phase II NeoCombi trial, patients received dabrafenib 150 mg twice daily plus trametinib 2 mg once daily for 12 weeks of neoadjuvant therapy before the resection of the tumor followed by adjuvant therapy for 40 weeks [54]. The response according to RECIST was 86%, and 46% achieved a complete response; the response according to pathological evaluation was 100%, and 49% had a pCR. The treatment was well-tolerated, with 29% of patients experiencing grade 3–4 events. The subsequent Neo-Trio clinical trial of neoadjuvant anti-PD-1 (pembrolizumab) in sequence with or concurrent with dabrafenib/trametinib in resectable Stage III *BRAF*V600-mutant melanoma patients showed that the pathological response rate (pRR of 80%) and the pathological complete response rate (pCR of 50%) were highest in the concurrent triple arm [55]. Noticeably, the initial induction course with dabrafenib/trametinib prior to anti-PD-1 did not improve the pathological response over anti-PD-1 monotherapy (pRR of 50% and 55%, respectively). However, treatment induction with BRAF/MEK inhibitors was short, and the number of patients included (n = 60) precluded any conclusions about the treatment efficacy.

### 2.4. Treatment Selection in BRAFV600-Mutant Patients with Active Brain Metastases

Patients with active symptomatic melanoma brain metastases (MBM) and concurrent corticosteroid use are largely excluded from clinical trials. As such, there is little evidence on the treatment efficacy of current treatments in this difficult-to-treat population with a high, unmet medical need. Combined treatment with ipilimumab/nivolumab and BRAF/MEK inhibitors has shown substantial intracranial activity in patients with active brain metastases, and the intracranial efficacy is principally equivalent to the extracranial treatment response [56,57,58]. To date, systemic treatment with ipilimumab/nivolumab yields the most durable responses in *BRAF*V600 patients with asymptomatic MBM, with substantial intracranial activity and similar outcomes to *BRAF*wt patients; in the updated results of the Checkmate 204 clinical trial, the 36-month OS was 73.0% (IQR 60.2–82.3) in asymptomatic patients [59].

There is limited data investigating the treatment efficacy of ICIs combined with BRAF/MEK inhibitors in patients with active MBM. In an exploratory analysis of the IMspire150 clinical trial, the time of the development of MBM in 491/514 patients enrolled in the study and without a history of MBM at baseline was numerically lower in patients receiving atezolizumab in combination with vemurafenib/cobimetinib compared with vemurafenib/cobimetinib, although the differences were small (25% vs. 21%, respectively) [60]. The TRICOTEL clinical trial is a phase 2 study that systematically investigated the treatment efficacy of anti-PD-L1 combined with BRAF/MEK inhibitors compared with BRAF/MEK inhibitors in patients with active MBM [61]. The study included 65 patients with *BRAF*V600-mutant melanoma, out of which 24 (37%) patients received corticosteroids or had symptomatic MBM at baseline. The primary endpoint of the study was the intracranial objective response rate (iORR) as assessed by an independent review committee (IRC). After an mFU of 9.7 months (IQR 6.3–15.0), the iORR in all patients was 42% (95% CI 29–54%) by IRC assessment and 51% (95% CI 38–63%) by investigator assessment. The median intracranial DoR was 7.4 months (95% CI 5.7–11.0), and the mPFS was 5.3 months (95% CI 3.8–7.2) by IRC. In patients with symptomatic MBM or corticosteroid use at baseline, the iORR was 46% (95% CI 26–67) by IRC and 58% (95% CI 37–78) by investigator assessment. The median intracranial DoR was 9.9 months (95% CI 4-8–12.7), and the mPFS was 5.4 months (95% CI 3.7–9.2) by IRC. In these patients, the administration of corticosteroids could be significantly reduced during the run-in cycle of combined BRAF/MEK inhibitors prior to the administration of atezolizumab. In the study population, the mOS was 13.7 months (95% CI 9.7–19.8), and in patients with symptomatic MBM or use of corticosteroids, the mOS was 16.6 months (95% CI 6.9–22.7).

The inclusion of patients with symptomatic MBM in the TRICOTEL study addressed a patient population with a per-definition poor prognosis and with an urgent need for the development of new therapeutic options. Although crossover comparisons should be made with caution, especially with regards to the different definition and inclusion of symptomatic MBM patients, these results compared to those reported in the Combi-MB study of dabrafenib/trametinib in *BRAF*V600 patients with symptomatic MBM [58]. In this study, 10 of 17 patients had an iORR of 59% (95% CI 33–82) by investigator assessment. After an mFU of 8.5 months (IQR 5.5–14.0), the mPFS was 5.5 months (95% CI 2.8–7.3), and the mOS was 11.5 months (95% CI 6.8–22.4). In addition, systemic treatment with anti-PD-1 resulted in an iORR of 6% and a 5-year OS of 13% in 20 patients with previously treated or untreated diseases and symptomatic MBM in the ABC clinical trial [57]. Overall, despite the marginal effect on the iORR, the addition of anti-PD-L1 to BRAF/MEK inhibitors in *BRAF*V600-mutant melanoma seemed to result in higher OS rates. Nonetheless, even with these promising results, combined treatment with ipilimumab/nivolumab continues to yield the most durable responses in patients with symptomatic MBM and has the longest mFUs [59]. In the Checkmate 204 clinical trial, combined treatment with ipilimumab/nivolumab resulted in an investigator-assessed iORR of 16.7% (IQR 3.6–41.4) in 18 patients with symptomatic MBM [56]. In the updated results, with an mFU of 34.3 months (IQR 14.7–36.4), the PFS rate at 36 months was 18.9% (95% CI 4.6–40.5), and the OS rate was 36.6% (14.0–59.8). Meanwhile, patients that achieved a response showed durable remission at 3 years [59]. Of note, 8/18 (44%) patients had the *BRAF*V600 mutant, but treatment outcomes were not reported in these patients. Lastly, in the ABC clinical trial, the iORR, the PFS and the OS were lower in patients treated with ipilimumab/nivolumab following progression on BRAF/MEK inhibitors [57]. Data on short induction treatment with BRAF/MEK inhibitors and an elective switch to ICIs are currently lacking.

## 3. Perspective

Three clinical trials, two phase 3 and one phase 2, have been designed to compare the efficacy of anti-PD-L1 in combination with BRAF/MEK inhibitors in BRAF*V600*-mutant melanoma [30,32,33]. Following preclinical data showing the underlying immunological effects of both BRAF and MEK inhibitors [14,15], as well as the increased efficacy of the triple combination in murine models [23], the addition of anti-PD-L1 to BRAF/MEK inhibitors in a clinical trial setting resulted in a prolonged PFS, but this difference was significant only in one study [30]. The recently published results of a meta-analysis evaluating the three randomized phase 2–3 trials could show, by summary estimate, an effective advantage on the PFS and the OS in the triplet combination [62]. However, whether this benefit on the OS rate supersedes the observed durable treatment effect in patients treated with ICIs remains to be elucidated as the data mature. Limited by their toxicity, the absence of anti-PD-1 as a comparative arm, as well as the marginal effect on the PFS rates and, in some patients, on the OS rates as well, the role of triple therapy in the treatment of advanced melanoma is debatable, and the long-term benefit of this treatment strategy remains to be seen as the data mature. To date, triple therapy has been investigated only in the first-line setting, and studies investigating the effect of triple therapy after treatment failure of adjuvant immunotherapy or targeted therapy are needed. Clinical trials evaluating the efficacy of triple combination in patients with anti-PD-1 refractory disease and the presence of MBM are underway (NCT02910700) [63].

On the contrary, combined treatment with ipilimumab/nivolumab has shown substantial treatment activity in *BRAF*V600-mutant melanoma. In the Checkmate 067 study, the absolute difference in the 6-year PFS and OS rates was greater for ipilimumab/nivolumab than nivolumab monotherapy in patients with *BRAF*V600-mutant disease (38% and 23%, respectively), whereas a smaller difference was shown in *BRAF*wt patients (34% and 32%, respectively), underlying that although responses to anti-PD-1 seem to be independent of the *BRAF* status, patients with *BRAF*V600-mutant melanoma seem to benefit more from ipilimumab/nivolumab [5]. Similarly, a recent study of 1764 patients with advanced melanoma from a nationwide registry confirmed the prolonged mPFS and mOS in *BRAF*-mutant melanoma compared with *NRAS*-mutant and double wild-type melanoma [64]. Prospective data on disease outcomes with sequential targeted therapy and ipilimumab/nivolumab support frontline treatment with ICIs over targeted therapy [42,45].

Despite preclinical data supporting the immunomodulatory effects of BRAF/MEK inhibitors in the tumor microenvironment, clinical and preclinical data suggest that this effect might be transient and that acquired resistance to BRAF/MEK inhibitors is suggestive of a cross-resistance to ICIs as well [43]. Notably, 8-week induction treatment with encorafenib/binimetinib in arm C of the SECOMBIT study resulted in a clinical benefit [42]. In addition, combined treatment with BRAF/MEK inhibitors can provide rapid symptomatic relief in patients with a high tumor burden and symptomatic disease at baseline [8]. As such, an initial induction course of targeted therapy followed by combined treatment with ipilimumab/nivolumab in patients with BRAF*V600*-mutant melanoma and symptomatic or rapid disease progression can be considered to achieve clinical stability and the reduction of the tumor volume. Nevertheless, the appropriate treatment duration of the induction treatment is not clear, and more systematic data are needed.

In patients with active MBM, ipilimumab/nivolumab yields the most durable responses to date [59]. However, patients with symptomatic MBM or with corticosteroid use at baseline represent a subpopulation with poor prognosis and of a high, unmet need. In the TRICOTEL study, combined treatment with anti-PD-L1, BRAF and MEK inhibitors resulted in intracranial efficacy and a reduction in the use of corticosteroids [61]. This significant reduction of the administration of corticosteroids was particularly due to the run-in cycle of combined BRAF/MEK inhibitors prior to the administration of atezolizumab. As such, patients with symptomatic brain metastases may profit from triple therapy, although long-term follow-up data are needed to confirm the durability of responses.

## 4. Conclusions

Overall, data from prospectively randomized clinical trials supported first-line treatment with ICIs and, in particular, ipilimumab/nivolumab in patients with *BRAF*V600-mutant melanoma. Still, patients at risk of rapid disease progression or death, as well as patients with symptomatic MBM, might profit from upfront treatment induction with BRAF/MEK inhibitors or from triple therapy. In the post hoc analysis of the COMBI–I study, patients with a high tumor load such as ≥3 metastatic sites seemed to profit more from the triple combination [32,36]. In the IMspire150 study, triple therapy showed a significantly better PFS in patients with elevated LDH as well as a numerical trend towards better outcomes in patients with a high tumor mutational burden [30]. As these results mature with further follow-up, the benefit of triple therapy on the OS rate, as well as the durability of responses, will be more evident. Lastly, these data highlight the imperative need for biomarkers for appropriate patient selection and the identification of those patients that would profit from triple combination.

## Figures and Tables

**Table 1:** Outcome data from clinical trials in *BRAF*V600 mutated melanoma.

	KEYNOTE-022	IMspire150	COMBI-i	TRICOTEL	CheckMate-067	COMBI-d/v
**Experimental Arm**	Pembrolizumab + Dabrafenib + Trametinib	Atezolizumab + Vemurafenib + Cobimetinib	Spartalizumab + Dabrafenib + Trametinib	Atezolizumab + Vemurafenib + Cobimetinib	Ipilimumab + Nivolumab	Dabrafenib + Trametinib
**Control Arm**	Dabrafenib + Trametinib	Vemurafenib + Cobimetinib	Dabrafenib + Trametinib	Atezolizumab + Cobimetinib	a. Ipilimumab b. Nivolumab	Dabrafenib
**Last Update**	22 June	22 June	22 June	22 August	21 November	19 June
**Phase**	II	III	III	II	III	III
**Number of Patients**	120	514	532	65 (*BRAF*V600), 15 (*BRAF*wt)	945	563
**Stage (AJCC)**	III–IV	IIIC–IV	IIIC–IV	IV (M1d)	III–IV	IIIC–IV
**Active Brain Metastases**	excluded	excluded	excluded	included (mandatory)	excluded	excluded
**Treatment Regimen Experimental Arm**	Pembrolizumab 200 mg IV every 3 weeks + Dabrafenib 150 mg p.o. 2x/d + Trametinib 2 mg p.o. 1x/d	**Cycle 1**: Vemurafenib 960 mg p.o. 2x/d for 28 days + Cobimetinib 60 mg p.o. 1x/d for 21 days (7 days off) **Cycle 2 onwards**: Atezolizumab 840 mg IV every 2 weeks + Vemurafenib 720 mg p.o. 2x/d for 28 days + Cobimetinib 60 mg p.o. 1x/d for 21 days (7 days off)	Spartalizumab 400 mg IV every 4 weeks + Dabrafenib 150 mg p.o. 2x/d + Trametinib 2 mg p.o. 1x/d	**Cycle 1**: Vemurafenib 960 mg p.o. 2x/d for 28 days + Cobimetinib 60 mg p.o. 1x/d for 21 days (7 days off) **Cycle 2 onwards**: Atezolizumab 840 mg IV every 2 weeks + Vemurafenib 720 mg p.o. 2x/d for 28 days + Cobimetinib 60 mg p.o. 1x/d for 21 days (7 days off)	**Cycle 1–4**: Nivolumab 1 mg/kg BW IV + Ipilimumab 3 mg/kg BW IV every 3 weeks **Cycle 5 onwards**: Nivolumab 3 mg/kg BW IV every 2 weeks	Dabrafenib 150 mg p.o. 2x/d + Trametinib 2 mg p.o. 1x/d
**Treatment Regimen Control Arm**	Placebo IV every 3 weeks + Dabrafenib 150 mg p.o. 2x/d + Trametinib 2 mg p.o. 1x/d	**Cycle 1**: Vemurafenib 960 mg p.o. 2x/d for 28 days + Cobimetinib 60 mg p.o. 1x/d for 21 days (7 days off) **Cycle 2 onwards**: Placebo IV every 2 weeks + Vemurafenib 960 mg p.o. 2x/d for 28 days + Cobimetinib 60 mg p.o. 1x/d for 21 days (7 days off)	Placebo IV every 4 weeks + Dabrafenib 150 mg p.o. 2x/d + Trametinib 2 mg p.o. 1x/d	Atezolizumab 840 mg IV every 2 weeks + Cobimetinib 60 mg p.o. 1x/d for 21 days (7 days off)	a. Placebo IV + Ipilimumab 3 mg/kg BW IV every 3 weeks for 4 cycles b. Placebo IV + Nivolumab 3 mg/kg BW every 3 weeks	Dabrafenib 150 mg p.o. 2x/d + Placebo p.o. 1x/d
**Median FU, months (95% CI)**	61.2 (50.7–67.5)	29.1 (1–54)	42.8	9.7 (6.3–15.0)	80.8 (74.0–86.3)	22 (0–76)
**ORR, % (95% CI)**	65 (52–77)	66.7	68.5 (62.6–74.1)	42 (29–54) ^1^	58 (53–64)	68
**Median DoR, months**	30.2 (14.1-NR)	21	NR (18.6-NR)	7.4 (5.7–11.0) ^1^	NR (21.0-NR) ^2^	12.9 (9.4–19.5)
**Progression-Free Survival, % (95% CI)**						
**Median PFS, months**	17.0 (11.3-NR)	15.1 (11.4–18.4)	16.2 (12.7–23.9)	5.3 (3.8–7.2) ^1^	16.8 (8.3–32.0) ^2^	11.0 (9.5–12.8)
**1-year PFS**	62 (48.1–73.5)	-	58	-	-	-
**2-year PFS**	47 (33.4–58.7)	-	44 (37–50)	-	-	31
**3-year PFS**	-	-	-	-	40	24
**4-year PFS**	-	-	-	-	-	21
**5-year PFS**	-	-	-	-	-	19
**HR for PFS**	0.46 (0.29–0.75)	0.79 (0.64–0.97)	0.82 (0.66–1.03)	-	a. 0.44 (0.31–0.62) b. 0.62 (0.44–0.89) ^2,3^	-
**Overall Survival, % (95% CI)**						
**Median OS, months**	46.3 (23.9-NR)	39.0	NR	13.7 (9.7–19.8)	NR (50.7-NR) ^2^	25.9 (22.6–31.5)
**1-year OS**	80 (67.5–88.1)	76.1	-	-	-	
**2-year OS**	63 (49.4–73.9)	62	67.7 (61.6–73.1)	-	-	52
**3-year OS**	-	-	60.1 (53.8–65.8)	-	68 ^2^	44
**4-year OS**	-	-	-	-	62 (52–71) ^2^	37
**5-year OS**	-	-	-	-	60 ^2^	34
**HR for OS**	0.64 (0.38–1.06)	0.84 (0.66–1.06)	0.80 (0.62–1.03)	-	a. 0.43 (0.30–0.60) b. 0.68 (0.46–1.02) ^2,3^	-
**Discontinuation of Treatment, n (%)**	43 (72)	115 (45)	181 (68)	63 (79)	-	-
**Biomarkers Associated with a Better Response to the Experimental Arm**	-	**PFS:** elevated LDH and negative PD-L1 status, trend towards high tumor mutational burden [≥10 mutations/Mb.] [35]	**PFS and OS:** bone and lung involvement at baseline, high tumor load (sum of lesion diameters ≥ 66 mm at baseline or metastatic sites ≥ 3, except for patients with liver metastases) with or without elevated LDH **PFS:** benefit in patients with increased NLR and sum of lesion diameters compared to placebo **OS:** ECOG PS 1, age ≥ 65 years, negative PD-L1 status [36,37]	-	-	-

Abbreviations*:* AE refers to adverse events, AJCC refers to the American Joint Committee on Cancer, BW refers to body weight, CI refers to confidence interval, DoR refers to duration of response, FU refers to follow-up, HR refers to hazard ratio, LDH refers to lactate dehydrogenase, mFU refers to median follow-up, NLR refers to neutrophil–lymphocyte ratio, NR refers to not reached, ORR refers to objective response rate, OS refers to overall survival, PD-L1 refers to programmed death ligand 1 and PFS refers to progression free survival. ^1^ ORR, DoR and PFS were in relation to intracranial activity, ^2^ specifically for patient cohort with *BRAF*V600 mutation; ^3^ the study was not powered to compare the three treatment arms.

**Table 2:** Toxicity profile of combination triple therapy vs. double therapy.

	Keynote-022				Imspire150				COMBI-i			
	Pembrolizumab + Dabrafenib + Trametinib		Dabrafenib + Trametinib		Atezolizumab + Vemurafenib + Cobimetinib		Vemurafenib + Cobimetinib		Spartalizumab + Dabrafenib + Trametinib		Dabrafenib + Trametinib	
	All	Grade ≥ 3	All	Grade ≥ 3	All	Grade ≥ 3	All	Grade ≥ 3	All	Grade ≥ 3	All	Grade ≥ 3
**Treatment-related AEs**	95%	58%	93%	25%	99%	79%	99%	73%	99%	55%	88%	33%
trAEs leading to dose reduction	27%		15%		-		-		88%	49%	73%	37%
trAEs leading to discontinuation	47%		20%		13%		16%		12%	9%	8%	5%
Use of immune-modulatory agents to manage AEs	-		-		63%		51%		-		-	
**Selected treatment-related AEs**												
Pyrexia	71.7%	10.0%	68.3%	3.3%	39%	1%	26%	1%	66%	5%	44%	3%
Chills	35.0%	0.0%	38.3%	1.7%	-	-	-	-	29%	0%	20%	<1%
Diarrhea	28.3%	3.3%	11.7%	0.0%	42%	2%	47%	3%	24%	<1%	15%	1%
Nausea	26.7%	0.0%	30.0%	0.0%	23%	<1%	26%	2%	24%	<1%	17%	0%
Skin rash	36.7%	5.0%	26.7%	0.0%	41%	9%	41%	9%	23%	3%	20%	<1%
CK elevation	-	-	-	-	16%	0%	12%	<1%	23%	7%	24%	5%
AST elevation	20.0%	6.7%	20.0%	3.3%	34%	13%	23%	9%	21%	3%	14%	1%
ALT elevation	20.0%	3.3%	16.7%	3.3%	30%	8%	20%	4%	18%	3%	13%	2%

Abbreviations: AE represents adverse events, ALT represents alanine aminotransferase, AST represents aspartate aminotransferase, and CK represents creatinine kinase.
